# Supplementary material for: The climate benefits from cement carbonation are being overestimated
Source: Nat Commun. 2024 Jun 6;15:4848. doi: 10.1038/s41467-024-48965-z (PMC11156638; doi:10.1038/s41467-024-48965-z)
Supplement: Supplementary file 1 — Supplementary Information [file 41467_2024_48965_MOESM1_ESM.pdf]

# The climate benefits from cement carbonation on being over-estimated

Elisabeth Van Roijen [1, †], Kati Sethares [1, †], Alissa Kendall [1], Sabbie A. Miller [1, ‡]

**Affiliations:**

[1]Department of Civil and Environmental Engineering, , 2001 Ghausi Hall, University of California, Davis, 95616

*Correspondence and requests for materials should be addressed to: sabmil@ucdavis.edu

[†] equal contributions

# The climate benefits from cement carbonation on being over-estimated

Elisabeth Van Roijen [1, †], Kati Sethares [1, †], Alissa Kendall [1], Sabbie A. Miller [1, ‡]

**Affiliations:**

[1]Department of Civil and Environmental Engineering, University of California, Davis

*Correspondence and requests for materials should be addressed to: sabmil@ucdavis.edu

[†] equal contributions

## Supplemental Information Contents

Supplementary Note ........................................ 2

1. Goal and scope of assessment ........................................ 2

2. Carbon Uptake Model Equations ........................................ 2

 2.1. Carbonation coefficient: $k_l$, $k_d$, $k_s$ ........................................ 2

 2.2. Carbonation depth: $d_l$, $d_d$, $d_t$ ........................................ 2

  2.2.1. Secondary Life ........................................ 2

 2.3. Volume of carbonated concrete during service life: $V_l$ ........................................ 3

 2.4. Maximum diameter of particles undergoing full carbonation: $D_0$, $D_l$ ........................................ 3

  2.4.1. Demolition ........................................ 3

  2.4.2. Secondary Life ........................................ 3

 2.5. Cumulative fraction of carbonated cement: $F_d$, $F_s$ ........................................ 3

  2.5.1. Demolition ........................................ 3

  2.5.2. Secondary Life ........................................ 3

 2.6. Cumulative mass of carbonated cement: $W_l$, $W_d$, $W_s$ ........................................ 3

 2.7. Cumulative carbon uptake: $C_l$, $C_d$, $C_s$ ........................................ 4

 2.8. Carbon uptake in mortar ........................................ 4

3. Dynamic modeling through time-adjusted warming potentials (TAWP) ........................................ 4

4. Measures to alter uptake and sensitivity analysis ........................................ 4

## Supplementary Note

# 1. Goal and scope of assessment

The goal of this study was to examine the carbonation effects, including time-adjusted warming potentials (TAWPs), to understand effects on cumulative radiative forcing and mechanisms to drive desired carbonation to mitigate climate burdens. The environmental impact analysis was conducted on a global scale, examining the cradle-to-grave (production, use and end-of-life) carbonation effects of concrete. Various concrete components were analyzed and incorporated to encapsulate the carbonation effects including cement content, supplemental cementitious (SCM's) materials, coarse aggregates, hydraulic lime and chemical admixtures. The impacts of carbon uptake were examined on a granular scale (per kg of cement) and on different regional scales.

Modeling the impacts of carbon uptake on a 'per kg' scale allowed for the examination of fluxes in GHGs associated with various production, use and demolition parameters. The impact of different parameter variations on the carbon uptake can identify which variations have the most potential to increase carbon uptake in concrete. Global and US cement production data was combined with concrete end use and longevity data to model the historic impacts of carbon uptake, projected future impacts, and asses the time-adjusted effect of regional cement consumption on the atmosphere. The regional models offer perspective of the historic performance of concrete in service and the rate of savings carbonation can achieve relative to different scales of cement consumption. The modeling efforts required to analyze the production statistics and examine carbonation effects are outlined in the subsequent sections.

# 2. Carbon Uptake Model Equations

Equations for calculating carbon uptake were adapted from the equations developed by Xi for their 2016 uptake model and adapted by Cao in 2020.[3,10] Only equations that vary from those presented by Xi and Cao are presented here. It is assumed that aggregates do not contribute to the carbon uptake potential of the concrete system.

## 2.1. Carbonation coefficient: $k_l$, $k_d$, $k_s$

The carbonation coefficient, k, is calculated using the same equations as Xi for all phases.[10]

## 2.2. Carbonation depth: $d_l$, $d_d$, $d_t$

The carbonation depth in the useful life and demolition phases are calculated using the same equation as Xi, a modified version of Fick's diffusion law.[10]

### *2.2.1. Secondary Life*

Carbonation depth in the secondary life is calculated as a total carbonation depth over the demolition and secondary life phases; see Equation 1.

$$d_t = k_s\sqrt{t_s} + k_d\sqrt{t_d} \tag{1}$$

Where $d_t$ is the total depth of carbonation over the demolition and secondary life phases, and $k_d$ and $k_s$ and $t_d$ and $t_s$ are the carbonation coefficients and phase durations of the demolition and secondary use phases, respectively. The durations may vary over time.

## 2.3. Volume of carbonated concrete during service life: $V_l$

During service life, the concrete carbonated can be most accurately estimated by volume. The volume equation used here is visually identical to Cao's, but the cement content, $C_c$, is a constant, and the cement mass and concrete thickness, $W_c$ and T, vary by end use market.[12] Both $W_c$ and the depth of carbonation, $d_l$, vary over useful life duration.

## 2.4. Maximum diameter of particles undergoing full carbonation: $D_0$, $D_1$

These values provide an estimate of the maximum diameter of particles that undergo carbonation during the active phase of the life cycle.

### *2.4.1. Demolition*

The maximum diameter of particles undergoing full carbonation in the demolition phase is represented by $D_0$ and the equation as defined by Xi.

### *2.4.2. Secondary Life*

The maximum diameter of particles undergoing full carbonation in both the demolition and secondary life phases is represented by $D_1$ and the equation as defined by Xi.

## 2.5. Cumulative fraction of carbonated cement: $F_d$, $F_s$

The fraction of carbonated cement is calculated assuming approximately spherical particles of cement are being carbonated. a and b are the range of particle size diameters discussed in 2.5.

### *2.5.1. Demolition*

The fraction of carbonated cement in the demolition phase, $F_d$, is defined using the same equations presented by Xi.

### *2.5.2. Secondary Life*

The fraction of cement carbonated over the demolition and secondary life, $F_s$, is defined by Equation 2. $F_s$ is a cumulative fraction.

$$F_s = \begin{cases} 100 - \dfrac{\int_a^b \frac{\pi}{6}(D-d_t)^3}{\int_a^b \frac{\pi}{6} D^3} & (a \geq D_1) \\ 100 - \dfrac{\int_{D1}^b \frac{\pi}{6}(D-d_t)^3}{\int_a^b \frac{\pi}{6} D^3} & (a < D_1 < b) \\ 100 & (b < D_1) \end{cases} \quad (2)$$

Where $d_t$ is the total carbonation depth over the demolition and secondary use phases and $D_1$ is the maximum diameter of particles undergoing full carbonation in the demolition and secondary use phases.

## 2.6. Cumulative mass of carbonated cement: $W_l$, $W_d$, $W_s$

The cumulative mass of carbonated cement in all phases are calculated using the same equations as Xi.[9]

## 2.7. Cumulative carbon uptake: $C_l$, $C_d$, $C_s$

The cumulative carbon uptake values in all phases are calculated using the same equations presented by Xi and Cao. Values of the constants $C_{clinker}$, $f_{CaO}$, $\gamma$, and $M_r$ are the same as presented by Xi et al.[10] The percentage lime in the clinker is assumed to be 65% as was assumed for calcination emissions in 2.2.

## 2.8. Carbon uptake in mortar

The cumulative carbon uptake in mortar applications was calculated using the same equations as Xi et al. [10] However, in order to simplify the number of equations used in the model, and reduce uncertainty associated with the results, it was assumed that no sides are rendered.

# 3. Dynamic modeling through time-adjusted warming potentials (TAWP)

The carbonation effects were examined with the incorporation of TAWP's. The TAWP's and resulting life-cycle impacts of concrete were calculated based on the equations 3 and 4, derived from Kendall et. al.[14] Cumulative radiative forcing (CRF) was calculated with and without time-adjusted warming impacts using equation 3 and data from IPCC on radiative forcing values of emission.[15] When calculating CRF without time-adjusted warming impacts, the y variable in equation 3 was set equal to zero.

$$CRF_{p,u,e} = \int_0^{AT-y} RF_{CO2}\, dt \tag{3}$$

$$Life\ Cycle\ TAWP = m_p * CRF_p + \sum_{i=1}^{useful\ life} m_{u,i} * CRF_{u,i} + \sum_{j=1}^{demolition\ phase\ length} m_{d,j} * CRF_{d,j} + \sum_{k=1}^{secondary\ life} m_{s,k} * CRF_{s,j} \tag{4}$$

The uptake of $CO_2$ in each year ($i$) of the useful life, end-of-life ($j$) and secondary life ($k$) were used to calculate total life cycle emissions. $CRF_{p,u,d,s}$ and $m_{p,u,d,s}$ refer to the CRFs and mass of emissions from production, use, demolition and secondary life respectively. In equation 3, $RF_{CO2}$ is the radiative forcing of $CO_2$, y is the year at which the emissions occur and AT is the analytical time horizon. Carbonation impacts were calculated using analytical time horizons of 20, 50 and 100 years.

The global warming potential impacts of 1 kg of cement was calculated assuming a use phase of 64 years, and demolition phase of 0.4 years and a secondary phase of 35 years. The amount of uptake associated with 0.4 years of demolition was utilized however it was modeled as occurring over 1 year in the TAWP calculations for simplicity. Cement was assumed to be exposed, uncoated and in an urban environment. During demolition, it was assumed that cement would be exposed, uncoated and in an industrial environment. Finally, given that a secondary-use for cement is likely to be some form of asphalt, cement in the secondary stage was assumed to be used in a road application and uncoated. The cumulative radiative forcing and TAWP data can be found in Supplementary Data 1, Sheet 15.

# 4. Measures to alter uptake and sensitivity analysis

A sensitivity analysis was conducted to examine the impact of various parameters on the resulting carbon uptake potential for 1 kg of cement. These parameters include demolition particle size, length of demolition stage, type and amount of SCM's, coating type, use-phase exposure conditions and location.

## 4.1 Scenario analysis summary table

The scenario analysis table focuses on the impacts associated with parameters in the life cycle of 1 kg of cement that can be managed or altered. These parameters include demolition particle size, length of demolition phase and type and amount of SCM's. Carbon uptake associated with secondary life was excluded from this analysis. During all life cycle phases the cement was assumed to be uncoated. During the use-phase, cement was assumed to be exposed in an urban environment whereas during demolition it was assumed to be in an industrial environment.

The demolition particle size diameter "base case" was 1-40 mm given the prevalence of this range of particles in literature.[16–19] However, carbon uptake was calculated assuming narrower ranges of 1-5, 1-10 and 1-30 mm to develop an understanding of the impact of demolition particle size on carbon uptake.

The length of time that the demolition particles are exposed can vary significantly and there is a lack of robust data regarding this process. Therefore, the demolition phase was assumed to be 1 day for the "base case" to highlight the "worst case scenario", but the impact of lengthening the demolition phase to 3 months and 1 year were also examined. Although 1 year is thought to be somewhat unrealistic in industry, the goal was to get an understanding of the full range of carbon uptake potentials.

The carbon uptake potentials for supplemental cementitious materials were obtained from a literature review by von Greve-Dierfeld *et al.* [13] Fly ash and natural pozzolans were assumed to have a maximum substitution potential of 50%. Limestone and silica fume were assumed to have maximum substitution potentials of 15%. Blast furnace slag was assumed to have a maximum substitution potential of 50%. In the mitigation summary table, SCM's were broken down into two main groups: cementitious (slag and limestone) and pozzolanic (fly ash, silica fume, natural pozzolans).

Supplementary Table 1. Results of scenario analysis for lifecycle emissions for 1 kg of cement.

| Process modification | total emissions | % reduction from base case GWP | TAWP (100 yr) | % reduction in TAWP |
|---|---|---|---|---|
| decreasing particle diameter to 1-30 mm | 0.85 | -7% | 0.78 | -3% |
| dec. particle diameter to 1-10 mm | 0.95 | -19% | 0.82 | -9% |
| dec. particle diameter to 1-10 mm and increase demo phase to 1 month | 0.81 | -2% | 0.76 | -1% |
| dec. particle diameter to 1-10 mm and increase demo phase to 3 months | 0.74 | 7% | 0.73 | 3% |
| dec. particle diameter to 1-10 mm and increase demo phase to 1 year | 0.69 | 13% | 0.71 | 6% |
| decreasing particle diameter to 1-5 mm | 0.94 | -19% | 0.82 | -9% |
| decreasing particle diameter to 1-5mm and demo phase 3 months | 0.72 | 9% | 0.72 | 4% |
| dec. particle diameter to 1-5 mm and increase demo phase to 1 year | 0.72 | 9% | 0.72 | 4% |
| lengthening demolition phase to 3 months | 0.71 | 11% | 0.72 | 5% |
| lengthening demolition phase to 1 year | 0.64 | 19% | 0.69 | 9% |
| 7.5% replacement with slag | 0.76 | 4% | 0.71 | 6% |
| 7.5% replacement with fly ash | 0.75 | 6% | 0.70 | 7% |
| 7.5% silica fume | 0.74 | 7% | 0.69 | 8% |
| 7.5% limestone | 0.76 | 4% | 0.71 | 6% |
| 25% replacement with slag | 0.63 | 21% | 0.57 | 24% |
| 25 % replacement with fly ash | 0.59 | 26% | 0.54 | 28% |
| 25% limestone | 0.60 | 24% | 0.55 | 26% |
| 50% replacement with slag | 0.44 | 44% | 0.39 | 48% |
| 50% replacement with fly ash | 0.40 | 50% | 0.35 | 53% |
| * base case: demolition phase 1 day, particle diameter 1 to 40 mm, no SCM | 0.79 | | 0.75 | |

### 4.2 Heat map sensitivity analysis

The heat maps highlight the impact of various use-phase parameters on carbon uptake potential such as coatings, exposure conditions and locations. Given that a secondary use phase of 35 years can result in substantial uptake but is not always guaranteed, the secondary phase was left out of the sensitivity analysis to highlight the impact of use-phase conditions. Further, since demolition is assumed to always take place outdoors, the cement was assumed to be exposed and in an industrial location during EoL. Crushed cement particles in the demolition phase were also assumed to be uncoated. The use phase was assumed to be 64 years and the demolition phase was assumed to be 0.4 years.

The heatmaps show the fraction of cement calcination emissions that are sequestered in a kg of cement for two locations: industrial and seaside. These locations were chosen because they

represent the locations with the highest (industrial) and lowest (seaside) carbon uptake potentials. The heat maps show the carbon uptake potential associated with and without 50% replacement with fly-ash. A replacement of 50% fly ash was chosen to highlight the range in carbon uptake potentials since fly ash has the highest carbon uptake potential of all the SCM's discussed in this study.

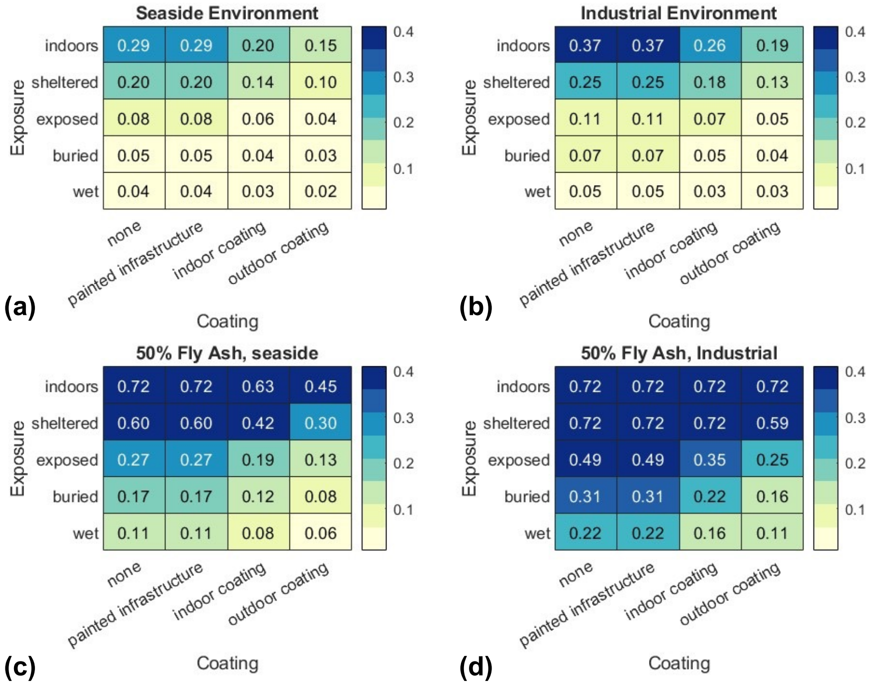


**Supplementary Figure 1.** Heat map results from sensitivity analysis for carbon uptake during use and end-of-life for 1 kg of cement. The color bar and associated numbers reflects the mass of $CO_2$ (in kg) taken up during use and end-of-life for 1 kg of cement. Two environmental conditions and two supplementary cementitious contents are presented in the four panels, a, b, c, and d.

## Supplementary References

1. Survey, U. S. G. Cement statistics, in Kelly, T.D., and Matos, G.R., comps., Historical statistics for mineral and material commodities in the United States: U.S. Geological Survey Data Series 140. *U.S. Geological Survey* http://minerals.usgs.gov/minerals/pubs/historical-statistics/ (2017).
2. Cao, Z., Shen, L., Løvik, A. N., Müller, D. B. & Liu, G. Elaborating the History of Our Cementing Societies: An in-Use Stock Perspective. *Environ. Sci. Technol.* **51**, 11468–11475 (2017).
3. Cao, Z. *et al.* The sponge effect and carbon emission mitigation potentials of the global cement cycle. *Nat. Commun.* **11**, 3777 (2020).
4. ERMCO. European Ready Mix Concrete Organization (ERMCO) Statistics 2016. (2017).
5. International Energy Agency (IEA). World Energy Outlook. (2023).
6. Gomez, D. R. . W. J. D. . A. B. B. . H. C. . M. G. . M. E. . N. L. N. . O.-E. B. . S. J. D. K. . T. K. . Q. R. . C. 2006 IPCC Guidelines for National Greenhouse Gas Inventories: Chapter 2: Energy: Stationary Combustion. in *I.P. on C.* (ed. Intergovernmental Panel on Climate Change) (2007).
7. GNR. Global Cement Database on $CO_2$ and Energy Information. http://www.wbcsdcement.org/GNR-2013/index.html (2016).
8. Marceau, M. L. . N. M. A. . V. M. G. *Life Cycle Inventory of Portland Cement Manufacture*. (2006).
9. van Oss, H. G. *Minerals yearbook: cement 2012*. https://www.usgs.gov/centers/national-minerals-information-center/cement-statistics-and-information (2015).
10. Xi, F. *et al.* Substantial global carbon uptake by cement carbonation. *Nat. Geosci.* **9**, 880–883 (2016).
11. Kapur, A., Keoleian, G., Kendall, A. & Kesler, S. E. Dynamic Modeling of In-Use Cement Stocks in the United States. *J. Ind. Ecol.* **12**, 539–556 (2008).
12. Pade, C. & Guimaraes, M. The CO2 uptake of concrete in a 100 year perspective. *Cem. Concr. Res.* **37**, 1348–1356 (2007).
13. von Greve-Dierfeld, S. *et al.* Understanding the carbonation of concrete with supplementary cementitious materials: a critical review by RILEM TC 281-CCC. *Mater. Struct.* **53**, 136 (2020).
14. Kendall, A. Time-adjusted global warming potentials for LCA and carbon footprints. *Int. J. Life Cycle Assess.* **17**, 1042–1049 (2012).
15. Intergovernmental Panel on Climate Change. *Climate change 2007: The physical science basis*. vol. 59 (2007).
16. Di Maria, F., Bianconi, F., Micale, C., Baglioni, S. & Marionni, M. Quality assessment for

recycling aggregates from construction and demolition waste: An image-based approach for particle size estimation. *Waste Manag.* **48**, 344–352 (2016).

17. Ulsen, C. *et al.* Production of recycled sand from construction and demolition waste. *Constr. Build. Mater.* **40**, 1168–1173 (2013).

18. Wu, S., Zhong, J., Zhu, J. & Wang, D. Influence of demolition waste used as recycled aggregate on performance of asphalt mixture. *Road Mater. Pavement Des.* **14**, 679–688 (2013).

19. Wang, X. *et al.* Optimized treatment of recycled construction and demolition waste in developing sustainable ultra-high performance concrete. *J. Clean. Prod.* **221**, 805–816 (2019).

20. Kikuchi, T. & Kuroda, Y. Carbon dioxide uptake in demolished and crushed concrete. *J. Adv. Concr. Technol.* **9**, 115–124 (2011).

21. Simion, I. M., Fortuna, M. E., Bonoli, A. & Gavrilescu, M. Comparing environmental impacts of natural inert and recycled construction and demolition waste processing using LCA. *J. Environ. Eng. Landsc. Manag.* **21**, 273–287 (2013).

22. Pradhan, S., Tiwari, B. R., Kumar, S. & Barai, S. V. Comparative LCA of recycled and natural aggregate concrete using Particle Packing Method and conventional method of design mix. *J. Clean. Prod.* **228**, 679–691 (2019).

23. Estanqueiro, B., Dinis Silvestre, J., de Brito, J. & Duarte Pinheiro, M. Environmental life cycle assessment of coarse natural and recycled aggregates for concrete. *Eur. J. Environ. Civ. Eng.* **22**, 429–449 (2018).
